# Military Tract Medical Association

**Published:** 1871-07

**Authors:** Herbert Judd

**Affiliations:** Secretary


### MILITARY TRACT MEDICAL ASSOCIATION.

The Sixth Annual Meeting of the Military Tract Medical Association was held in the Masonic Hall, at Galesburg, Tuesday, June 13th. M. Reece, of Abingdon, President, occupied the chair. Herbert Judd, of Galesburg, Secretary.

Forty members were present, representing eight counties. During the morning session the following new officers were elected:

*President*—S. P. Breed, of Princeton.

*Vice-President*—John M. Moose, of Galesburg.

*Secretary and Treasurer*—Herbert Judd, of Galesburg.

*Board of Censors*—J. K. Crawford, of Monmouth; M. Reece, Abingdon; Wm. Hamilton, Galesburg.

The address delivered by M. Reece, the retiring president, was voted to be spread upon the records.

The principal business was reports from the following standing committees.

*Essayists*—E. L. Phillips, Galesburg; M. A. McClelland, Knoxville.

*Surgery*—Wm. Hamilton, Galesburg; J. N. Todd, Kewanee; Geo. W. Crossley, Princeton.

*Practice of Medicine*—Hiram Nance, Kewanee; W. L. Cuthbert, Monmouth; John P. McClanahan, Norwood.

*Materia Medica and Therapeutics*—S. P. Breed, Princeton; H. Marshall, Monmouth; J. V. McCutchen, Norwood.

*Obstetrics and Diseases of Women and Children*—S. K. Crawford, Monmouth; W. D. Stirling, Ionia; John W. Hensley, Yates City.

*Ophthalmology*—L. S. Lambert, Galesburg.

The cases of surgery reported by Dr. Hamilton were interesting; one case, an amputation of the arm near the shoulder, in which one of the ligatures (silk) still remained, the operation being performed fifteen weeks since. In the discussion that followed, Dr. Hamilton, of Monmouth, recommended from his practice the silver wire ligature, cut off short and left in the stump. He had always had good results and healing by the first intention.

Upon the practice of medicine considerable matter of a general character was presented.

Dr. Wm. Byrns, of New Windsor, reported a man who took 200 grs. of powd. opium. He was some distance away, and when found was completely comatose. This case was successfully treated by emetics, strong coffee, cold affusion, and active motion of the patient.

President Breed read an extensive and valuable report upon therapeutics.

The Committee on Obstetrics reported several cases of difficult labor, one with a fatal result.

Dr. L. S. Lambert read a lengthy paper upon "Anomalies of Refraction." This paper, together with the various reports, were referred to the Committee on Publication. Under miscellaneous business considerable interesting matter was discussed. Drs. Nance, S. M. Hamilton, Webster, Cuthbert, and Hilbert, presented some new rules in regard to membership and dues, which were adopted by the Association.

Dr. A. V. F. Gilbert was appointed by the President as Committee upon Necrology. The deaths of Dr. H. C. Graham, of New Windsor, and Dr. Geo. Gillis, of North Prairie, were reported and referred to this committee.

The meeting was thoroughly business-like, and seemed profitable to all. Galesburg was voted as the place for the next meeting.

Upon the motion of Dr. Hiram Nance, the Association voted that the Secretary forward a report of this meeting to the *Chicago Medical Examiner* for publication. Dr. H. S. Hurd, of Galesburg, moved, which was voted that the report be also forwarded to the *Chicago Medical Journal* for publication.

The Association then adjourned to meet at Galesburg the second Tuesday in January.

HERBERT JUDD, *Secretary.*

